# Tandem duplication of chromosomal segments is common in ovarian and breast cancer genomes

**DOI:** 10.1002/path.4042

**Published:** 2012-08

**Authors:** David J McBride, Dariush Etemadmoghadam, Susanna L Cooke, Kathryn Alsop, Joshy George, Adam Butler, Juok Cho, Danushka Galappaththige, Chris Greenman, Karen D Howarth, King W Lau, Charlotte K Ng, Keiran Raine, Jon Teague, David C Wedge, Australian Ovarian Cancer Study Group, Xavier Caubit, Michael R Stratton, James D Brenton, Peter J Campbell, P Andrew Futreal, David DL Bowtell

**Affiliations:** 1 Cancer Genome Project, Wellcome Trust Sanger InstituteHinxton, Cambridge, UK; 2 Cancer Genomics and Genetics, Peter MacCallum Cancer CentreMelbourne, Australia; 3 Department of Pathology, University of MelbourneAustralia; 4 Cancer Research UK Cambridge Research InstituteCambridge, UK; 5 Department of Biochemistry and Molecular Biology, University of MelbourneParkville, Australia; 6 Hutchison/MRC Research Centre and Department of Pathology, University of CambridgeUK; 7 Developmental Biology Institute of Marseille-Luminy (IBDML), UMR 6216, CNRS-Inserm-Université de la MéditerranéeFrance; 8 Sir Peter MacCallum Department of Oncology, University of MelbourneAustralia

**Keywords:** ovarian cancer, structural rearrangements, *TSHZ3*

## Abstract

The application of paired-end next generation sequencing approaches has made it possible to systematically characterize rearrangements of the cancer genome to base-pair level. Utilizing this approach, we report the first detailed analysis of ovarian cancer rearrangements, comparing high-grade serous and clear cell cancers, and these histotypes with other solid cancers. Somatic rearrangements were systematically characterized in eight high-grade serous and five clear cell ovarian cancer genomes and we report here the identification of > 600 somatic rearrangements. Recurrent rearrangements of the transcriptional regulator gene, *TSHZ3,* were found in three of eight serous cases. Comparison to breast, pancreatic and prostate cancer genomes revealed that a subset of ovarian cancers share a marked tandem duplication phenotype with triple-negative breast cancers. The tandem duplication phenotype was not linked to *BRCA1/2* mutation, suggesting that other common mechanisms or carcinogenic exposures are operative. High-grade serous cancers arising in women with germline *BRCA1* or *BRCA2* mutation showed a high frequency of small chromosomal deletions. These findings indicate that *BRCA1/2* germline mutation may contribute to widespread structural change and that other undefined mechanism(s), which are potentially shared with triple-negative breast cancer, promote tandem chromosomal duplications that sculpt the ovarian cancer genome. Copyright © 2012 Pathological Society of Great Britain and Ireland.

## Introduction

Somatically acquired structural genomic rearrangements are common in most solid cancers and are especially so in ovarian cancer 1. Ovarian cancer is a collective term for a number of distinctly different histotypes and tumours of varying degrees of malignancy 2. The most common epithelial ovarian cancer histotypes include serous, clear cell, mucinous and endometrioid. Amongst these, high-grade serous cancers (HGSCs) are the most common, accounting for approximately 70% of all cases of invasive epithelial ovarian cancer and the majority of deaths.

Increasingly comprehensive genomic analyses of HGSCs and clear cell cancers are providing insights into pathways of transformation and the molecular determinants of response to therapy. Our previous gene expression profiling of HGSCs identified four molecular subtypes 3, which are associated with different clinical outcomes 3, 4. A signalling pathway involving *MYCN, LIN28B* and *LET7* is associated with one of the four subtypes 4, suggesting that specific pathway activation may drive the development and behaviour of one or more HGSCs molecular subtypes. DNA copy number analyses have shown that gains and losses are particularly frequent in HGSCs 5, 6, with a high level of genomic disorganization apparent in most cancer samples. A recent detailed genomic analysis of 500 HGSCs by the TCGA consortium identified frequently amplified and deleted regions of the HGSC genome 1. Approximately 50% of HGSCs had defects in the BRCA1/2 pathway, either through germline or somatic mutation or methylation of pathway members.

Clear cell cancers are associated with endometriosis 7, and have been recently found to harbour somatic mutations in the *ARID1A* gene in approximately 50% of tumours 8, 9. The patterns of gene expression and copy number change seen in clear cell ovarian cancer are distinct from HGSCs, and frequently involve amplification and over expression of cytokines including IL6, receptor tyrosine kinases and other downstream signalling components 10, 11. The gene expression profiles of ovarian clear cell cancers are similar to renal and uterine clear cell tumours 12. Favourable responses have been observed in a small number of ovarian clear cell patients to sunitinib 10, a drug with considerable activity in renal clear cell cancer.

Next-generation DNA sequence analysis is providing an unprecedented level of information about the cancer genome, identifying new mutations 9, 13, 14, the impact of mutagens 15, 16 and novel processes that sculpt the cancer genome 17. Here we used paired-end DNA sequencing to seek novel gene fusions and characterize structural changes in ovarian cancer samples, comparing and contrasting HGSCs and clear cell genomes.

## Materials and methods

### Patient samples and ethics

Tumour samples and clinical data were obtained from women enrolled in the Australian Ovarian Cancer Study (http://www.aocstudy.org). All participants provided written informed consent and Human Research Ethics Committee approval was obtained at the Peter MacCallum Cancer Centre (Queensland Institute of Medical Research, University of Melbourne, Australia) and all participating hospitals for the study. Further clinical data, information on biospecimens and microarray analysis are described in the Supplementary methods (see Supporting information).

### Next-generation sequencing

Next-generation sequencing and structural variant analysis was carried out as described previously 18. Briefly, 37 bp paired-end reads generated on the Illumina GA2 were aligned to the human reference genome (hg19) using BWA. Rearrangement breakpoints were called when two or more discordantly mapped read pairs supported the same underlying event. Breakpoints were classified according to the relative orientations and insert sizes of the read pairs into those suggesting deletion, translocation, inversion or tandem duplication (insertion). Candidate breakpoints were confirmed as somatic by PCR on both tumour and matched normal DNA and mapped to base pair resolution by capillary sequencing. Breakpoints were classified according to the relative orientations and insert sizes of the read pairs into those suggesting deletion, translocation, inversion or tandem duplication (insertion). Rearrangements were further classified based on integration with copy number data to include amplicon junctions, fold-back inversions and genomic shards 19.

### Validation of gene rearrangements

cDNA from total RNA for sequenced samples and validation samples was synthesized using M-MLV reverse transcriptase (Promega), as described previously 20. Endpoint RT–PCR was performed according to standard protocols using Thermo-Start DNA polymerase (ThermoScientific). Products were resolved by agarose gel electrophoresis and samples with visible products (for *CCNY/CREM* and *ATP9B* rearrangements only) were subjected to a second independent PCR reaction with alternate primer sets. No products were confirmed by a second PCR reaction except for control samples, where rearrangements were initially identified. Gene expression of *TSHZ3* was measured by quantitative reverse-transcription PCR (qRT–PCR) normalized to *ACTB* and *HPRT1* control genes and median *C*_T_ (threshold cycle) values obtained across eight serous samples, using a SYBR Green qPCR assay on the 7900HT Fast Real-Time PCR system (Applied Biosystems, Foster City, CA, USA). RNA was treated with DNase I (Promega) prior to cDNA synthesis to eliminate background amplification of genomic DNA. Primer details are given in Table S4 (see Supporting information).

### Germline and somatic analysis of *BRCA* pathway dysfunction

Complete germline sequencing of *BRCA1* and *BRCA2* and multiplex ligation-dependent probe amplification (MLPA) for detection of large chromosomal aberrations were undertaken in the National Association of Testing Authorities (NATA) accredited Molecular Pathology Laboratory at the Peter MacCallum Cancer Centre. Detailed methods are provided elsewhere (Alsop *et al*, in press). Somatic mutations in tumour samples were screened by high-resolution melt (HRM) analysis of all coding exons of *BRCA1* and *BRCA2*, as described by others 21. Gene promoter methylation of tumour DNA was assessed for *BRCA1, FANCF* and *PALB2* using methylation-sensitive HRM, as described previously 22. Reported percentage of methylated allele present in tumour DNA was estimated by comparison to a panel of control samples. No promoter methylation of *FANCF* or *PALB2* was identified in the 13 samples screened, and only one sample showed promoter methylation of *BRCA1* (see Supporting information, Table S1).

## Results

**Figure 1:**
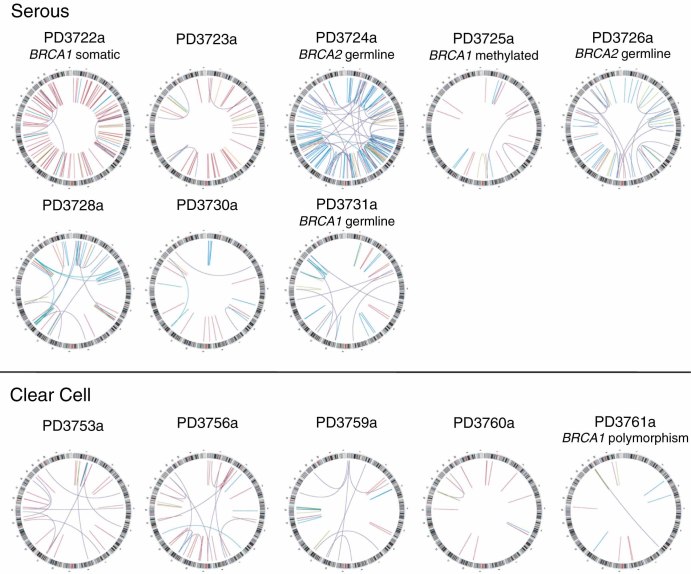
Genomic rearrangements of 13 ovarian cancer genomes. The chromosomes of the reference genome are drawn around the circumference of each circos plot 42. Rearrangements are represented by lines linking somatically acquired breakpoints: orange, fold back inversions; light blue, one or both end map to an amplicon; purple, translocations; green, inversions; red, tandem duplications; dark blue, deletions. *BRCA1/2* mutation status or mechanism of inactivation indicated

To identify DNA rearrangements in ovarian cancer genomes, we carried out paired end sequencing of tumour DNA from 13 ovarian tumour samples (eight HGSCs and five clear cell; see Supporting information, Table S1). A total of 1.1 × 10^9^ 37 bp read pairs generated 7 × 10^10^ base pairs that could be assigned uniquely to the reference genome equating to 4.3–16.5-fold physical coverage per sample. Putative structural rearrangements were identified from incorrectly mapping read pairs 18, 23 and 634 confirmed somatically acquired DNA rearrangements were identified across 13 ovarian cancers (range 13–150/sample), with base pair sequence level resolution of 598 individual breakpoints (94%; see Supporting information, Table S2). Each genome displayed a different spectrum of unique somatically acquired rearrangements (Figure 1), with varying proportions of rearrangements in each class (Table 1).

**Table 1:** Breakpoint frequencies in 13 ovarian cancer cases illustrating correlations with *BRCA1*/*2* status and histotype

Sample	Histotype	BRCA status	Totalbreakpoints	Translocations (%)	Deletions (%)	Tandem duplications (%)	Amplicons (%)	Inversions (%)	Fold-back	Shards (%)
PD3722a	Serous	Somatic (*BRCA1*)	114	4	7	***71***	7	8	2	2
PD3723a	Serous	Wt	37	8	3	***68***	3	11	8	0
PD3724a	Serous	Germline (*BRCA2*)	150	24	***51***	13	3	6	1	3
PD3725a	Serous	Methylated (*BRCA1*)	19	11	16	***68***	0	5	0	0
PD3726a	Serous	Germline (*BRCA2*)	60	22	***42***	17	0	18	0	2
PD3728a	Serous	Wt	75	9	19	16	***36***	5	11	4
PD3730a	Serous	Wt	21	10	29	***48***	10	0	5	0
PD3731a	Serous	Germline (*BRCA1*)	30	23	***27***	20	17	7	3	3
PD3753a	Clear cell	Wt	31	32	3	***58***	0	6	0	0
PD3756a	Clear cell	Wt	39	23	10	***62***	3	3	0	0
PD3759a	Clear cell	Wt	30	27	17	***30***	0	20	0	7
PD3760a	Clear cell	Wt	15	7	7	***73***	0	7	0	7
PD3761a	Clear cell	Polymorphism of likely low clinical significance	13	8	23	***38***	0	23	8	0

Categories with the highest proportion of breaks for each tumour are in bold italics.

By classifying tumours according to the dominant class of rearrangement, three distinct mutation profiles were observed in HGSCs. In four of the HGSCs, half or more of the chromosomal rearrangements involved tandem duplications (median 410 kb), including one case with 81 tandem duplication events detected (PD3722a). For three HGSCs, deletions were the dominant rearrangement class, with the remaining serous cancer sample having a genome dominated by junctions within amplicons (Table 1). The three cases showing a high frequency of deletions had either a germline *BRCA1* or *BRCA2* mutation (PD3724a, PD3726a and PD3731a) and the deletions in these cases were small (median 3.2 kb) when compared to deletions in cases without germline *BRCA1*/*2* mutations (median 288 kb). Over-representation of deletions in *BRCA1/2* mutant compared to wild-type cases as a proportion of all rearrangements was statistically significant (*p* < 3.91 × 10^−11^ by a Poisson regression test). Two HGSCs cases with somatic alteration of *BRCA1*, either through point mutation or promoter hypermethylation (PD3722a and PD3725a, respectively), did not show the deletion/translocation phenotype. Fewer rearrangements were observed in ovarian clear cell cancers compared with HGSCs (Table 1), consistent with our previous copy number analysis 10. The types of rearrangement present within each genome were more evenly distributed between the classes in the clear cell histotype. Tandem duplications were the dominant class in all five clear cell cases, but relatively high proportions of translocations, deletions and inversions were also seen (Table 1). Statistical analyses utilizing a generalized linear model (GLM) approach indicated that the occurrence of deletions (*p* = 0.0053) and amplicons (*p* = 0.010) was significantly different between clear cell and HGSCs cases. Further, the overall *p* value for the probability that the distributions differ (from the log likelihood, when compared against the null model of no interaction between cancer type and rearrangement class distribution) was 3.13 × 10^−6^.

We have previously observed high proportions of tandem duplications in a subset of breast cancer genomes 23. Given the apparent similarity in pattern and distribution of this class of rearrangement, we undertook a comparative analysis of ovarian and other solid cancers for which data was available. Although HGSCs and clear cell cancers are considered to have different aetiologies and patterns of somatic mutation, we considered them as a single group for the purposes of the analysis, as tandem duplications were found in both histotypes. We compared 634 ovarian cancer rearrangements identified in this study, with 994 rearrangements identified in primary breast cancers 23, 352 rearrangements in pancreatic cancers 19 and 464 rearrangements recently reported in an analysis of seven prostate cancer genomes 24. Comparing the proportions of each class of rearrangement in each cancer type revealed significant differences (χ^2^*p* value for effects of interaction between cancer type and rearrangement type = 1.88 × 10^−68^). There were significantly more tandem duplications in breast and ovarian cancers compared to pancreatic and prostate cancers (*p* = 1.6 × 10^−12^). Amplified fold back inversions were much more common in pancreatic than breast or ovarian cancers (*p* = 4.94 × 10^−04^).

**Figure 2:**
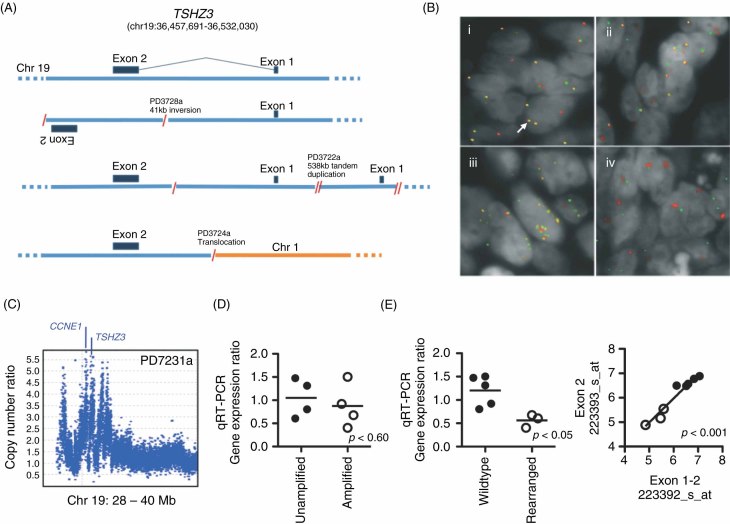
(A) Wild-type *TSHZ3* locus (top) and schematics of the three rearrangements identified by next-generation sequencing. (B) Tissue microarray images of four cases with varying *TSHZ3* rearrangements: (1) loss of the 5′ end of *TSHZ3* with example of locus without rearrangement indicated by a white arrow; (ii) balanced break; (iii) amplification; and (iv) breakage with amplification of the 3′ end of *TSHZ3*. Green, BAC probes RP11-280H11 and RP11-241C16 (5′ *TSHZ3*); red, RP11-161K19 and RP11-164O11 (3′ *TSHZ3*). (C) Affymetrix SNP 6.0 copy number data for tumour sample PD7231a, showing multiple focal amplifications incorporating *CCNE1* and *TSHZ3*. (D) Scatter plot of *TSHZ3* expression by gene copy number status, where amplification is > three copies. (E) Scatter plot showing *TSHZ3* gene expression by qRT–PCR (left) and gene expression microarray (right) in rearranged tumours (open circles) compared to samples without gene rearrangement (closed circles)

In total, 371 (59%) of the somatic rearrangement breakpoints identified in this study fell in or within 10 kb of gene ‘footprints’ (ie within Ensembl gene coordinate boundaries), with 16 gene footprints disrupted in more than one sample (see Supporting information, Table S3). While these mostly occurred in genes with large genomic footprints, suggesting that a proportion are likely passenger events, several warrant further consideration. *TSHZ3*, a homeobox transcription factor that has been implicated in ureter formation 25 and development of respiratory neurons 26, was the most commonly disrupted gene, occurring in 3/8 HGSC samples. The breakpoints (caused by translocation, inversion or tandem duplication) all lie in the only intron and map within 29 kb of each other (Figure 2A). The gene is approximately 74 kb in length and we have not found it deleted in other cancer types previously (unpublished data).

Rearrangement of *TSHZ3* was further explored by two-colour FISH analysis of 90 HGSCs samples on tissue microarrays (TMA), which included three cases (PD3722a, PD3724a, PD2728a) identified in the rearrangement screen. In total, 11/90 HGSC cases were found to have rearrangements involving the *TSHZ3* locus. The small tandem duplication in PD3722a was not detectable on TMA FISH, whilst the translocation in PD3724a was readily confirmed. The inversion in PD3728a was not discernable on FISH; however, the locus was amplified. In the remaining cases, there were two main patterns of rearrangement, split roughly equally between amplification and rearrangement breaks in the gene, including two cases with apparently balanced breaks, confirming *TSHZ3* as recurrently rearranged (Figure 2B).

Assessing the importance of *TSHZ3* in ovarian cancer is confounded by its proximity to *CCNE1*, mapping 1.45 Mb telomeric on 19q12 (Figure 2C). *CCNE1* is known to be amplified and operative as a cancer gene in serous ovarian cancer 27, and the breakpoints in *TSHZ3* could be collateral to amplification involving *CCNE1*. To further evaluate this, those TMA cases with *TSHZ3* breakpoints and available DNA (*n* = 10) were evaluated using Affymetrix SNP 6.0 gene arrays for evidence of independence from *CCNE1* amplification. Five of 10 cases had high-level amplification of *CCNE1* with associated gain and breaks in *TSHZ3*, a frequency substantially above the 15–20% seen in unselected cases 20. Sample PD7231a shows a complex pattern of copy number change, including multiple, focal, high-level amplifications incorporating both *CCNE1* and *TSHZ3* (Figure 2C). In this case, *TSHZ3* and *CCNE1* are on separate segments, although they are presumably co-amplified. Of the five cases without *CCNE1* amplification, four had evidence for low-level copy-number gain (copy numbers 3 or 4) of both *TSHZ3* and *CCNE1*, with *TSHZ3* breakpoints detectable on SNP array in two of the cases; PD7229a and PD7730a both had balanced rearrangements on FISH and were not detectable on SNP array, as expected. In the remaining case, PD3722a, there was no copy number gain for *TSHZ3* other than the small tandem duplication of the locus, whereas *CCNE1* had a single copy gain. These data suggest that *TSHZ3* rearrangement can occur at least in some instances independently of *CCNE1* amplification. Additionally, Affymetrix SNP 6.0 data obtained from the TCGA ovarian cancer study (see Methods) was also evaluated for independent breakpoints affecting *TSHZ3* in the absence of CCNE1 amplification. This analysis revealed five cases with evidence for *TSHZ3* breakpoints apparently unrelated to *CCNE1* gain or amplification.

Quantitative RT–PCR analysis showed *TSHZ3* expression was not associated with gene amplification (Figure 2D); however, there was evidence of reduced expression of *TSHZ3* in rearranged cases compared with HGSCs without disruption of the locus (Figure 2E). We also compared gene expression by microarray, using probe sets detecting transcripts across exon 1 and 2 boundaries (223392_s_at) and within exon 2 only (223393_s_at). No differences between probe sets were detected in rearranged versus wild-type samples, suggesting that there is no aberrant transcription of short transcripts (Figure 2E). To further identify potential fusion transcripts we employed 5′ RACE across all eight sequenced samples. We were, however, only able to detect wild-type products (data not shown). If novel transcripts were generated in the rearranged samples, it is possible that low expression may have limited their detection.

These findings suggest that if *TSHZ3* is contributing to ovarian cancer, it may be through down-regulation or loss of function. However, arguing against this is the lack of truncating mutations reported in the recently released data from whole-exome sequencing of more than 250 serous ovarian cancers, where only two missense and two silent somatic mutations were identified 1.

We examined *TSHZ3* gene expression further in two large independent datasets of HGSCs (see Supporting information, Figure S1). Although rearrangement of *TSHZ3* is associated with reduced gene expression, we found that a high level of *TSHZ3* was associated with shorter progression-free survival only in the AOCS dataset (*p* < 0.04) 3. In both AOCS and TCGA 1 cohorts, high *TSHZ3* expression was associated with the C1 molecular subtype, which is characterized by intense tumour stromal and epithelial–mesenchymal transition gene expression signatures and poor clinical outcome.

Non-identical tandem duplications of approximately 1 and 0.5 Mb were identified in 2/8 HGSCs samples that disrupted the gene encoding Rho GTPase activating protein 6 (*ARHGAP6*) on the X chromosome (see Supporting information, Table S3). These two cases were also rearranged for *TSHZ3. ARHGAP6* functions as a GAP for RhoA GTPase, which has been implicated in cell motility, and angiogenesis and has been reported to be up-regulated in multiple cancers, including serous ovarian carcinomas 28. Normally, *ARHGAP6* would serve to limit the activity of RhoA by activating the intrinsic GTPase activity resulting in conversion to the inactive GDP-bound form of the enzyme 29. The locus encodes multiple splice forms, but no significant expression differences were discernable in the rearranged versus non-rearranged cases.

Amongst the other genes broken, *ARID1A*, a member of the SWI/SNF chromatin remodelling complex, was found to be presumptively inactivated by a ∼130 kb inversion on chromosome 1 in the clear cell case, PD3753a. Frequent inactivating point mutations and a single rearrangement were recently reported in clear cell ovarian cancer 8, 9, consistent with the inactivating rearrangement identified here. Indeed, four of the five cases reported here were also included in Wiegand *et al* 9, with one case, PD3761a, having frameshift mutation in *ARID1A*. Also, *ARID1A* has been previously implicated in other human cancers 30, 31 and recently found to be mutated recurrently in clear cell renal cell carcinoma 32. Additionally, breakpoints in *PPP2R2B*, a component of the regulatory subunit B of serine/threonine phosphatase 2 (PP2A) involved in cell growth and division, were found in two samples (one serous and one clear cell tumour). Mutations in the PP2A regulatory subunit A component, *PPP2R1A*, have previously been shown in ovarian clear cell cancers 8.

Although all 13 HGSCs and clear cell cases had rearrangements, no recurrent gene fusions were detected amongst the discovery set of samples. Nine single instances of in-frame fusion genes and five in-frame internally rearranged genes were identified (Table 2), all occurring in HGSCs. To investigate whether any of these potential gene fusions and internally rearranged genes were transcribed, PCR assays were designed to the exons surrounding the genomic breakpoints and RNA from the relevant cases was assayed by reverse transcription (RT)–PCR. Products were successfully amplified for 7/9 gene fusions but none of the internally rearranged genes, indicating that a substantial proportion of in-frame fusions were expressed. RT–PCR product sequence was verified by conventional sequencing.

**Table 2:** In-frame gene fusions and internal gene rearrangements

Sample	5′ Gene	5′ Gene Accession No.	Exons in predicted fusion gene	3′ Gene	3′ Gene Accession No.	Exons in predicted fusion gene	Expressed?	Class of rearrangement
PD3722a	HGS*	NM_004712.3	1–8	SLC16A5*	NM_004695.2	6–7	Yes	Tandem duplication
PD3722a	*CCNY*	NM_145012.4	1–11	*CREM*	NM_181571.1	4–14b	Yes	Other intrachromosomal
PD3723a	*C2orf67*	NM_152519.2	1–3	*MARCH4*	NM_020814.2	3–4	Yes	Other intrachromosomal
PD3723a	*MRPL43*	NM_176792.1	1–5	*SH3PXD2A*	NM_014631.2	5–14	No	Tandem duplication
PD3723a	*C11orf41*	NM_012194.1	1–18	*DNAJC24*	NM_181706.4	4–5	Yes	Other intrachromosomal
PD3724a	*AGGF1*	NM_018046.3	1	*SCAMP1*	NM_004866.4	3–9	Yes	Deletion
PD3726a	*NDUFA11*	NM_175614.2	1–2	*STX17*	NM_017919.2	5–8	Yes	Translocation
PD3731a	*MAP3K1*	NM_005921.1	1–13	*SIRPA*	NM_001040022.1	9–10	Yes	Translocation
PD3760a	*SV2B*	NM_014848.3	1–8	*CRTC3*	NM_022769.3	11–15	No	Shard (TD)
PD3722a	*PHACTR1*	NM_030948.1	Duplication of exons 3 & 4				No	Tandem duplication
PD3722a	*SGCZ*	NM_139167.2	Duplication of exons 2 & 3				No	Tandem duplication
PD3726a	*CLSTN2*	NM_022131.2	Deletion of exon 2				No	Other intrachromosomal
PD3728a	*ATP9B*	NM_198531.3	Duplication of exons 12–15				ND	Tandem duplication
PD3760a	*THSD4*	NM_024817.2	Duplication of exons 6 & 7				No	Tandem duplication

* Alternative splicing of the fusion transcript yields both in-frame and out-of-frame products. ND = not determined.

In order to detect recurrence of novel rearranged transcripts in ovarian cancer, we screened cDNA from an independent validation set of 109 HGSCs for the same exon joining events for six of the in-frame fusion genes found in HGSCs cases (*CCNY*/*CREM, NDUFA11*/*STX17, AGGF1*/*SCAMP1, MAP3K1*/*SIRPA, C2orf67*/*MARCH4, C11orf41*/*DNAJC24*) and one of the in-frame internally rearranged transcripts (*ATP9B*). We were unable to detect expression of any of the fusion transcripts in the validation cohort. We next looked for co-expression of *CCNY/CREM* in our previous analysis of ovarian tumours 3. We reasoned that oncogenic fusion transcripts would likely show gene over-expression and preselection of cases may increase the chance of detecting recurrent low-frequency fusions. We identified 25 samples from 215 HGSCs (independent of the 109-sample validation set) that showed high expression of both *CREM* and *CCNY* (expression above median + 0.5× median absolute deviation [MAD]), representing a statistically significantly higher proportion than would be expected by chance (*p* < 0.05). From these, RNA was available for 15 samples and analysed for expression of the *CCNY/CREM* fusion. No fusion products were detected in the selected samples, suggesting that if co-expression was the result of rearrangement/fusion, breakpoints varied from the originally defined boundaries.

## Discussion

The landscape of rearrangements in HGSCs and clear cell ovarian cancers is characterized by a substantial proportion of cases, showing a predominance of tandem duplications, overlapping a phenotype previously shown in breast cancer 23. In addition, there is evidence for a deletion/translocation phenotype potentially linked to germline *BRCA1*/*2* mutations, with a high prevalence of small deletions being particularly marked in *BRCA2* null tumours. Somatic missense mutation and hypermethylation of *BRCA1* was not, however, associated with the same mutation phenotype. Therefore, somatic events are unlikely to be functionally equivalent to germline truncating mutations if abrogation of *BRCA1/2* function is directly linked to the phenotype. Overall comparison between four different tumour types suggests that multiple different mechanisms are operative in the reconstruction of cancer genomes, likely involving as-yet unidentified exposures and/or genome maintenance defects.

The observed high frequency of tandem duplications in the ovarian cancer samples, especially HGSCs cases, adds to previous data suggesting molecular similarities between triple-negative and HGSCs 33, 34. Of note, the tandem duplication phenotype was present in both histological subtypes of ovarian cancer. This is of interest, as these subtypes show marked differences in cancer gene mutations found 35, expression clustering 10 and response to therapy. Given the apparently different histogenesis 36, our findings suggest convergent acquisition of the tandem duplication phenotype during oncogenesis. Further, tandem duplication was not associated with germline *BRCA1* or *BRCA2*, suggesting that the phenotype is attributable to a yet to be identified common pathogenetic mechanism.

A recent study by Ng *et al* reported on the presence of a tandem duplicator phenotype in HGSCs 37. Genome paired-end sequencing of PEO1/PEO4 cell lines derived from a *BRCA2* mutation carrier showed interchromosomal breakpoints and small deletions consistent with homologous recombination (HR) deficiency. In contrast, the PEO14/PEO23, *BRCA* wild-type cell lines had intact HR and a tandem duplicator phenotype. Using SNP copy number data, a ‘tandem duplicator-like’ (TD-like) pattern was inferred and used to estimate the phenotype frequency in the TCGA dataset. TD-like copy number aberrations were reported to occur in 12.8% of HGSCs and, consistent with our findings, these were mutually exclusive to *BRCA1/2* carrier mutations.

We found no evidence of recurrent fusion genes in our study. While less common than in haematological malignancies and sarcomas, recurrent gene fusions have been identified in other solid tumours, such as *TMPRSS2–ERG* in prostate 38 and *EML4–ALK* in non-small cell lung cancers 39. Recently, Salzman *et al* reported on the identification of the novel *ESRRA–C11orf20* gene fusion in serous ovarian cancer, using an ultra-high-throughput RNA sequencing approach 40. Recurrence was reported in approximately 15% of cases (*n* = 67); however, we saw no evidence of this rearrangement in our screen. Identification of *ESRAA–C11orf20* fusions may have been limited by a low frequency of recurrence and the small number of serous tumours analysed in our study.

*TSHZ3* was identified as a target of recurrent breakage in ovarian cancer. Gene expression analysis suggests that if *TSHZ3* is contributing to pathogenesis, it may be through loss of function or simply down-regulation. Inactivation of *TSHZ3 in vivo* shows clear gene dosage effects, leading to haploinsufficiency, neonatal lethality and reduced heterozygote pup size compared to wild-type siblings 26. Additionally, *TSHZ3* has recently been shown to be among the most down-regulated genes in breast and prostate cancer, suggesting likely relevance in various tumour types 41. Additional work is warranted to clarify mechanisms of deregulation and the role of *TSHZ3* in ovarian cancer.

## Data access

Next-generation read datasets are available under managed access from the European Genome-Phenome Archive (EGA; http://www.ebi.ac.uk/ega/). Microarray data GEO submission in progress.

## Supporting Information on the Internet

The following supporting information may be found in the online version of this article:

Figure S1 Analysis of *TSHZ3* gene expression in AOCS and TCGA datasets.

Table S1 Sample details.

Table S2 Summary of somatic rearrangements found in 13 ovarian cancers.

Table S3 Breakpoints in known genes.

Table S4 Primer sequences.

Table S5 Tumour cell content predicted by ASCAT and PICNIC.
