# Supplementary figures and images for: Tandem duplication of chromosomal segments is common in ovarian and breast cancer genomes
Source: J Pathol. 2012 Aug;227(4):446–55. doi: 10.1002/path.4042 (PMC3428857; doi:10.1002/path.4042)

Supplementary Figure 1

A. AOCS (high-grade serous cases)

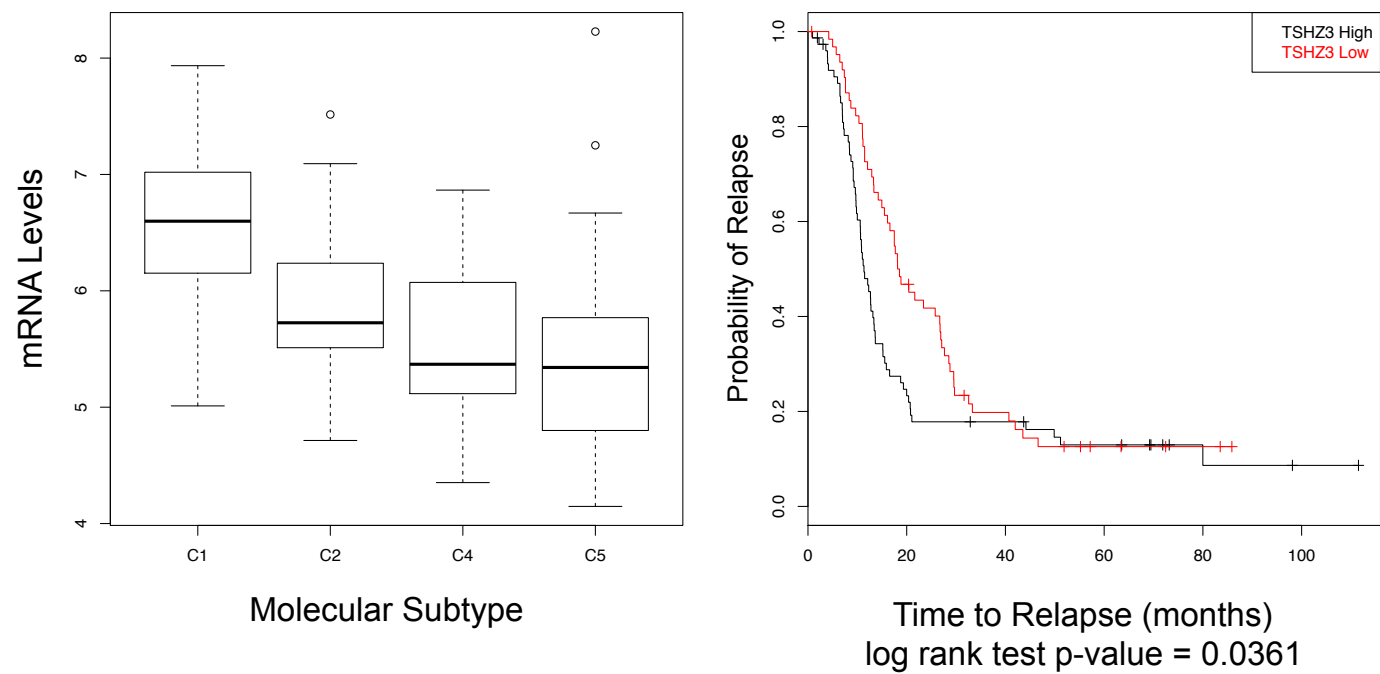

B. TCGA (high-grade serous cases)

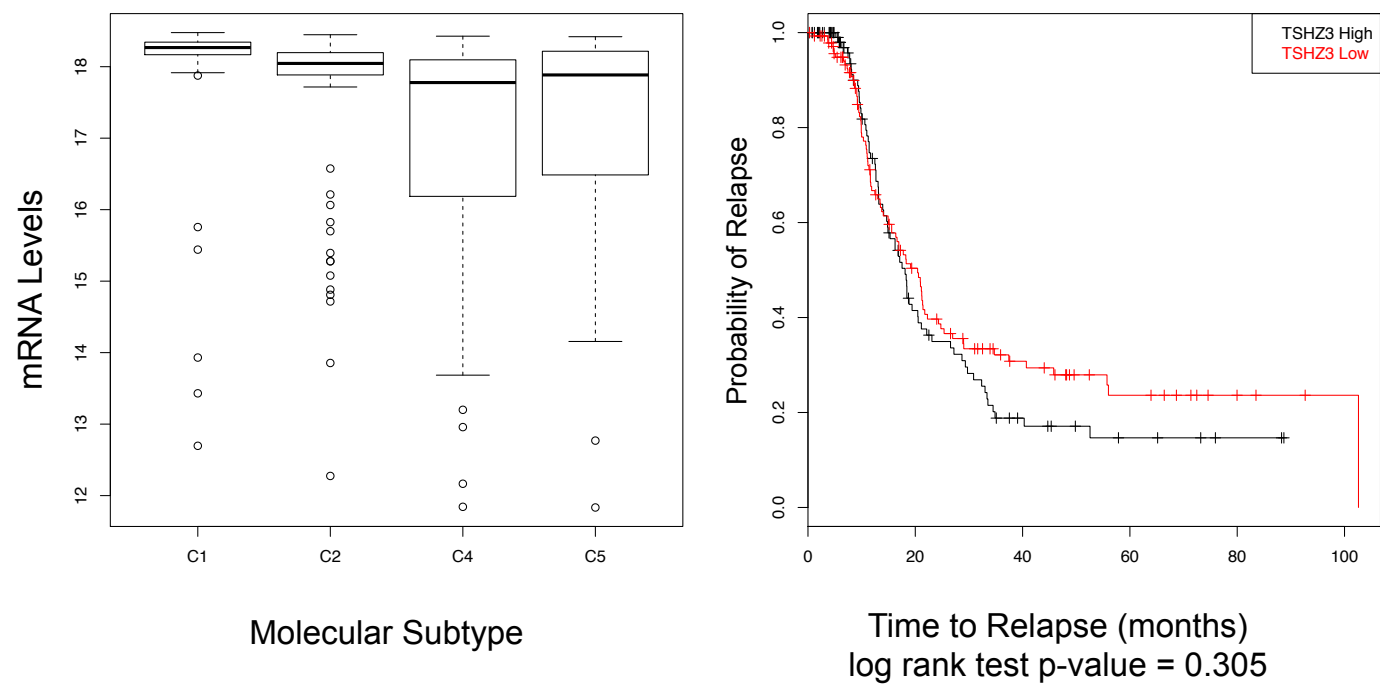

Supplement: Figure S1 — Analysis of TSHZ3 gene expression in AOCS and TCGA datasets. [file path0227-0446-sd1.pdf]
