# Supplementary material for: Tandem duplication of chromosomal segments is common in ovarian and breast cancer genomes
Source: J Pathol. 2012 Aug;227(4):446–55. doi: 10.1002/path.4042 (PMC3428857; doi:10.1002/path.4042)
Supplement: Table S5 — Tumour cell content predicted by ASCAT and PICNIC. [file path0227-0446-sd6.doc]

| **Sample ID** | **ASCAT** | **PICNIC** |
| --- | --- | --- |
| PD3722a | 49% | 62% |
| PD3723a | 59% | 65% |
| PD3724a | 67% | 66% |
| PD3725a | 25% | 70% |
| PD3726a | 67% | 64% |
| PD3728a | 65% | 72% |
| PD3730a | 52% | 68% |
| PD3731a | 57% | 65% |
| PD3753a | 72% | 65% |
| PD3756a | 73% | 66% |
| PD3759a | 57% | 63% |
| PD3760a | 43% | 68% |
| PD3761a | 34% | 68% |

**Table S5.** Tumour cell content predicted by ASCAT and PICNIC
