# Supplementary material for: Tandem duplication of chromosomal segments is common in ovarian and breast cancer genomes
Source: J Pathol. 2012 Aug;227(4):446–55. doi: 10.1002/path.4042 (PMC3428857; doi:10.1002/path.4042)
Supplement: Supplementary file 7 [file path0227-0446-sd7.doc]

**Supplementary Methods**

Australian Ovarian Cancer Study Patient Samples

All patients underwent laparotomy for diagnosis, staging and tumor de-bulking. Tumor material for genomic studies was taken at the time of primary surgery prior to the administration of chemotherapy. All women received standard treatment including carboplatin and paclitaxel and no patient received neoadjuvant treatment (Supplementary Table 1). Surgical staging was assessed in accordance with Federation Internationale des Gynaecologistes et Obstetristes (FIGO) classification.

Biospecimens for Genomic Analysis

Hematoxylin and eosin (H&E) stained sections from fresh-frozen tumor material was assessed for tumor content by a pathologist at the Peter MacCallum Cancer Centre. Samples contained 60-90% (median 80%) neoplastic cells and DNA was extracted from whole tumor tissue (Supplementary Table 1). Genomic DNA was extracted according to standard protocols with a DNeasy kit (Qiagen) and quantified by spectrophotometry (Nanodrop). Total RNA from whole tumor samples was isolated by phenol-chloroform extraction (Invitrogen) and Qiagen RNeasy column purification (Qiagen). Quality of RNA was assessed using a Bioanalyzer 2100 (Agilent).

Tissue microarrays (TMA) were constructed using formalin-fixed paraffin-embedded (FFPE) tissue blocks used either for diagnostic purposes or collected for research. Representative epithelial tumor components were marked by a pathologist on adjacent H&E sections and matched to the corresponding area on the diagnostic/research block. Two 0.6 mm cores from each section were then sampled by needle punch biopsy and re-embedded in a paraffin-processed agarose matrix recipient block. TMA fluorescence *in situ* hybridization (FISH) was carried out according to the method of Chin et al. [[1]](#_ENREF_29) using BAC probes RP11-280H11 and RP11-241C16 for the 5’ end of *TSHZ3* and RP11-161K19 and RP11-164O11 for the 3’ end of *TSHZ3*.

Gene Expression and Copy Number Microarrays

Genome-wide copy number data was generated using the cytogenetics copy number assay protocol and Affymetrix SNP 6.0 microarrays according to standard methods (Affymetrix, Santa Clara). Data was processed using ASCAT [3] and PICNIC [4] to derive SNP copy number and estimate tumour cell content (Supplementary Table 5). RNA samples were prepared for gene expression analysis using the GeneChip 3’ IVT Express Kit and hybridized onto U133 plus 2.0 microarrays according to the manufacturer’s protocol (Affymetrix, Santa Clara) and as previously described [[2]](#_ENREF_3).

We obtained the level 1 raw data (NCBI/TCGA project number 2459) for microarrays run on the Affymetrix SNP 6.0 platform for the TCGA study [5]. We excluded 18 arrays that failed TCGA quality control and a batch of 47 arrays that had poor signal to noise ratio. In six cases, donors had more than one sample profiled and we retained only the sample with the best quality in each case. In total, we retained 454 tumor samples for further analysis. We extracted normalized log ratio using Affymetrix Power Tools.

**References**

1. Chin SF, Daigo Y, Huang HE, Iyer NG, Callagy G, Kranjac T *et al.* A simple and reliable pretreatment protocol facilitates fluorescent in situ hybridisation on tissue microarrays of paraffin wax embedded tumour samples. Molecular pathology : MP 2003;**56**:275-279.

2. Tothill RW, Tinker AV, George J, Brown R, Fox SB, Lade S *et al.* Novel molecular subtypes of serous and endometrioid ovarian cancer linked to clinical outcome. Clinical cancer research : an official journal of the American Association for Cancer Research 2008;**14**:5198-5208; DOI 10.1158/1078-0432.CCR-08-0196.

3. Van Loo P, Nordgard SH, Lingjaerde OC, Russnes HG, Rye IH, Sun W *et al.* Allele-specific copy number analysis of tumors. Proc Natl Acad Sci U S A 2010;**107**:16910-16915; DOI 1009843107 [pii]

10.1073/pnas.1009843107.

4. Greenman CD, Bignell G, Butler A, Edkins S, Hinton J, Beare D *et al.* PICNIC: an algorithm to predict absolute allelic copy number variation with microarray cancer data. Biostatistics 2010;**11**:164-175; DOI kxp045 [pii]

10.1093/biostatistics/kxp045.

5. TCGA. Integrated genomic analyses of ovarian carcinoma. Nature 2011;**474**:609-615; DOI 10.1038/nature10166.
