# Supplementary material for: Tandem duplication of chromosomal segments is common in ovarian and breast cancer genomes
Source: J Pathol. 2012 Aug;227(4):446–55. doi: 10.1002/path.4042 (PMC3428857; doi:10.1002/path.4042)
Supplement: Supplementary file 8 [file path0227-0446-sd8.doc]

**<Supporting information>**

**Figure S1.** Analysis of *TSHZ3* gene expression in: (A) AOCS dataset [3]; and (B) TCGA dataset [1]. Box plots (left) depicting *TSHZ3* gene expression in HGSCs stratified by Tothill *et al*, molecular subtype. Kaplan–Meier plot (right) of progression-free survival for HGSCs patients stratified by high (median + 0.5 × median absolute deviation [MAD]) or low (median – 0.5 × [MAD]) *TSHZ3* mRNA level
